# The influence of CONSORT on the quality of reporting of randomised controlled trials: an updated review

**DOI:** 10.1186/1745-6215-12-S1-A47

**Published:** 2011-12-13

**Authors:** Lucy Turner, David Moher, Larissa Shamseer, Laura Weeks, Jodi Peters, Amy Plint, Douglas G Altman, Kenneth F Schulz

**Affiliations:** 1 Ottawa Hospital Research Institute, Ottawa, Canada; 2 Children’s Hospital of Eastern Ontario, Ottawa, Canada; 3 Centre for Statistics in Medicine, University of Oxford, Oxford, UK; 4 FHI360, North Carolina, USA

## Background

The Consolidated Standards of Reporting Trials (CONSORT) Statement was developed in response to concerns about the quality of reporting of randomized controlled trials (RCTs). The checklist is an evidence-based minimum set of recommendations for reporting RCTs, intended to facilitate the complete and transparent reporting of RCTs and aid in their critical appraisal and interpretation. In 2006, Plint and colleagues published a systematic review examining the effectiveness of CONSORT for improving the reporting of RCTs in journals that have formally endorsed the guidance (i.e. at minimum recommend that authors use CONSORT) [1]. Despite poor methodology of some included studies, use of CONSORT was found to be associated with improvement in the quality of reporting of RCTs.

## Objective

To update Plint et al.’s systematic review assessing the influence of the CONSORT Statement’s checklist (2001) on the quality of reporting of RCTs.

## Methods

Conventional systematic review methods employed in the original review by Plint et al. have been implemented. The search for new studies spanned August 2005 – March 2010. Two independent reviewers screened studies for eligibility; extraction and validity assessment of studies were conducted by a single reviewer and a second reviewer performed verification. Reporting quality was assessed by comparing the proportion of RCTs adhering to individual CONSORT items or a total sum score between comparison groups.

## Results

Of 2896 possibly relevant studies, 53 reports of 50 quasi-experimental studies have been included, compared to 8 in the earlier review. In total these studies assessed adherence to CONSORT in 16,222 RCTs. When comparing reporting in RCTs of CONSORT endorsing journals with CONSORT non-endorsing journals; 25 of 27 outcomes yield higher relative reporting of these items in endorsing journals, of which 7 were statistically significant. The largest positive effect, across 16 studies, showed that reporting of allocation concealment was 81% greater in CONSORT endorsing journals (RR = 1.81, 95%CI 1.37 to 2.40). There is no evidence to suggest that CONSORT endorsement has a detrimental influence on the quality of reporting of RCTs.

## Impact

Despite the questionable validity of the included studies, this updated review provides stronger evidence suggesting that CONSORT is associated with improved reporting of RCTs. This information is helpful to authors, peer-reviewers and journal editors when deciding whether to recommend or enforce the use of CONSORT.
